# Proteome Changes Induced by Iprodione Exposure in the Pesticide-Tolerant *Pseudomonas* sp. C9 Strain Isolated from a Biopurification System

**DOI:** 10.3390/ijms251910471

**Published:** 2024-09-28

**Authors:** Pamela Donoso-Piñol, Gabriela Briceño, Joseph A. M. Evaristo, Fábio C. S. Nogueira, Heidi Schalchli, María Cristina Diez

**Affiliations:** 1 Programa de Doctorado en Ciencias de Recursos Naturales, Universidad de La Frontera, Temuco 4780000, Chile; p.donoso01@ufromail.cl; 2 Departamento de Ciencias Químicas y Recursos Naturales, Universidad de La Frontera, Temuco 4780000, Chile; 3 Centro de Excelencia en Investigación Biotecnológica Aplicada al Medio Ambiente (CIBAMA-BIOREN), Universidad de La Frontera, Temuco 4780000, Chile; heidi.schalchli@ufrontera.cl; 4 Laboratorio de Proteómica, LADETEC, Instituto de Química, Universidad Federal de Rio de Janeiro, Rio de Janeiro 22775-000, Brazil; joseph.am.evaristo@gmail.com (J.A.M.E.); fabiocsn@ufrj.br (F.C.S.N.); 5 Departamento de Ingeniería Química, Universidad de La Frontera, Temuco 4780000, Chile

**Keywords:** pesticide biodegradation, pesticide tolerance, proteome, iprodione-degrading bacteria, iprodione, metabolism

## Abstract

Iprodione is a pesticide that belongs to the dicarboximide fungicide family. This pesticide was designed to combat various agronomical pests; however, its use has been restricted due to its environmental toxicity and risks to human health. In this study, we explored the proteomic changes in the *Pseudomonas* sp. C9 strain when exposed to iprodione, to gain insights into the affected metabolic pathways and enzymes involved in iprodione tolerance and biodegradation processes. As a result, we identified 1472 differentially expressed proteins in response to iprodione exposure, with 978 proteins showing significant variations. We observed that the C9 strain upregulated the expression of efflux pumps, enhancing its tolerance to iprodione and other harmful compounds. Peptidoglycan-binding proteins LysM, glutamine amidotransferase, and protein Ddl were similarly upregulated, indicating their potential role in altering and preserving bacterial cell wall structure, thereby enhancing tolerance. We also observed the presence of hydrolases and amidohydrolases, essential enzymes for iprodione biodegradation. Furthermore, the exclusive identification of ABC transporters and multidrug efflux complexes among proteins present only during iprodione exposure suggests potential counteraction against the inhibitory effects of iprodione on downregulated proteins. These findings provide new insights into iprodione tolerance and biodegradation by the *Pseudomonas* sp. C9 strain.

## 1. Introduction

The pesticide iprodione (IPR) (3-(3,5-dichlorophenyl) N-isopropyl-2,4-dioxoimidazoli-18 dine-1-carboxamide) is a member of the dicarboximide fungicide family, created to combat various agronomical pests such as grey mould disease and onion white rot caused by different types of fungi, particularly *Botrytis* [1,2]. Previous studies have investigated the toxic effects of IPR, revealing its impact on sexual development in mice, neurobehavioral alterations, and its potential carcinogenicity in humans [3,4,5].

Due to this established toxicity, effective management of IPR residues is necessary. One in-farm method for controlling pesticide water residues is through pesticide biopurification systems (BPSs), which rely on their biomixture components (wheat straw, peat, and soil) for the removal of pesticides via biodegradation processes by microorganisms [6]. Accordingly, various types of soil and BPS bacteria have been identified and reported as capable of degrading IPR. These microorganisms include genera such as *Arthrobacter*, *Achromobacter*, *Microbacterium*, and *Pseudomonas*, among others [7,8,9]. These bacterial strains possess the enzymatic machinery necessary to biodegrade IPR, making them of interest for deciphering the metabolic pathways of IPR biodegradation.

A metabolic pathway has been suggested for the degradation of IPR, beginning with its conversion to N-(3,5-dichlorophenyl)-2,4-dioxoimidazolidine (metabolite I), followed by the formation of 3,5-dichlorophenylurea acetic acid (metabolite II), and ultimately leading to the production of 3,5-dichloroaniline (3,5-DCA) [10,11]. Recent studies have identified a gene called ipaH, which has an affinity for aromatic amino compounds, as having potential for the initial degradation of IPR [12]. Furthermore, an *Achromobacter* sp. C1 strain has been shown through proteomic analysis to undergo significant changes in its protein expression during the biodegradation of IPR, principally amidohydrolases, enzymes reported to be capable of degrading IPR due to their C-N affinity.

This study aimed to gain insights into the metabolic pathways and enzymes involved in the bacterial biodegradation process. Understanding the metabolic processes associated with the degradation of pesticides facilitates the construction of biotechnological tools that detoxify these compounds, which are hazardous to human health and the environment. To achieve this, we performed a gel-free comparative proteomic analysis, comparing the proteomes of *Pseudomonas* sp. C9 grown in the presence and absence of the fungicide IPR. *Pseudomonas* sp. C9 is an IPR-degrading bacterium isolated from a BPS used to treat pesticides [7] and has been reported to achieve high degradation rates of IPR, with more than 91% removal within 48 h, and exhibited a twofold increase in growth under IPR treatment [7,13] making strain C9 a suitable candidate for an in-depth comparative proteomic analysis under IPR degradation. The results of this study could contribute to understanding resistance mechanisms and provide valuable information for enhancing bioremediation strategies, ultimately reducing the environmental impacts of pesticides.

## 2. Results

### 2.1. Overall Results of Pseudomonas sp. C9 Protein Expression in Response to IPR Exposure

**Figure 1:**
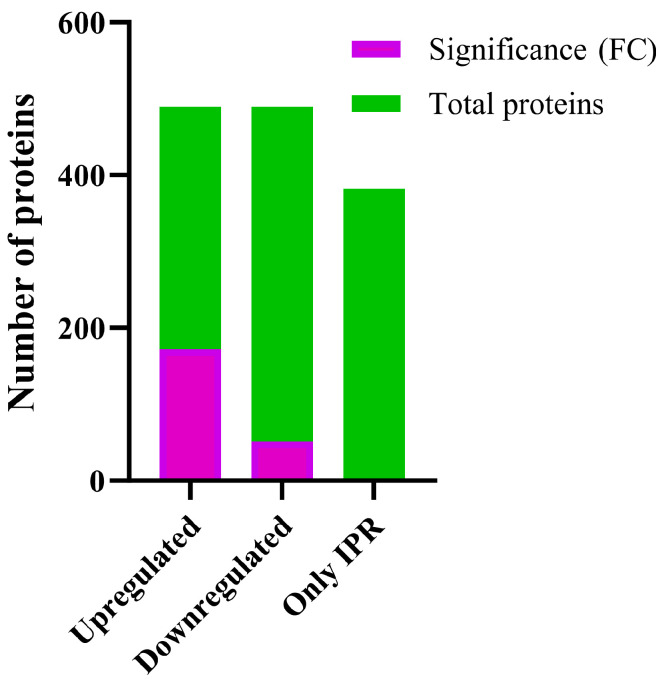
Differential expression of proteins of strain *Pseudomonas* sp. C9 in response to IPR treatment. Total: total number of proteins. Significance (FC): the threshold for differential expression was set as follows: upregulated proteins (≥1.21-fold change) and downregulated proteins (≤−1.21-fold change). Only IPR: proteins identified only in IPR treatment.

The proteomic composition of *Pseudomonas* sp. C9 when exposed to a concentration of 50 mg L^−1^ of IPR was analysed using the gel-free method. The mass spectrometric data were then analysed to identify the differentially expressed proteins compared to the untreated control. Out of a total of 1472 identified proteins, 978 proteins exhibited variations in expression, and 489 proteins were upregulated. Nonetheless, only 223 exhibited significant expression variations (≥1.21 and ≤−1.21-fold change) in the IPR-treated sample compared to the untreated control (Figure 1). Additionally, 382 proteins were solely present upon treatment with IPR. These findings revealed distinct protein profiles of *Pseudomonas* sp. C9 exposed to IPR, indicating the activation of specific proteins and pathways that contribute to the degradation and tolerance of the pesticide IPR (Table S1).

### 2.2. Identification and Analysis of Differentially Expressed Proteins in Response to IPR Treatment

**Figure 2:**
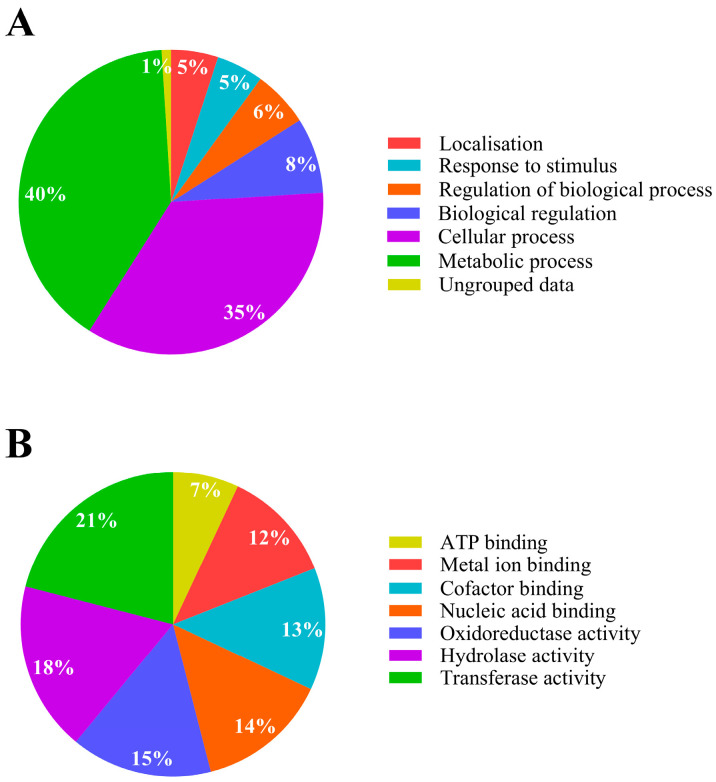
Metabolic activity analysis of *Pseudomonas* sp. C9 in response to iprodione. (**A**) Bacterial biological process; (**B**) bacterial molecular function. Modified from Blast2GO software [14].

For the strain *Pseudomonas* sp. C9, analysis using Blast2GO software version 4.1 showed that the biological processes involved in IPR degradation and tolerance corresponded primarily to metabolic and cellular processes, representing 40% and 35%, respectively (Figure 2A). Additionally, based on molecular function, the proteins identified in strain C9 were mainly involved in transferase activity (21%) and hydrolase activity (18%), followed by other functions (Figure 2B).

### 2.3. Upregulated Proteins

**Figure 3:**
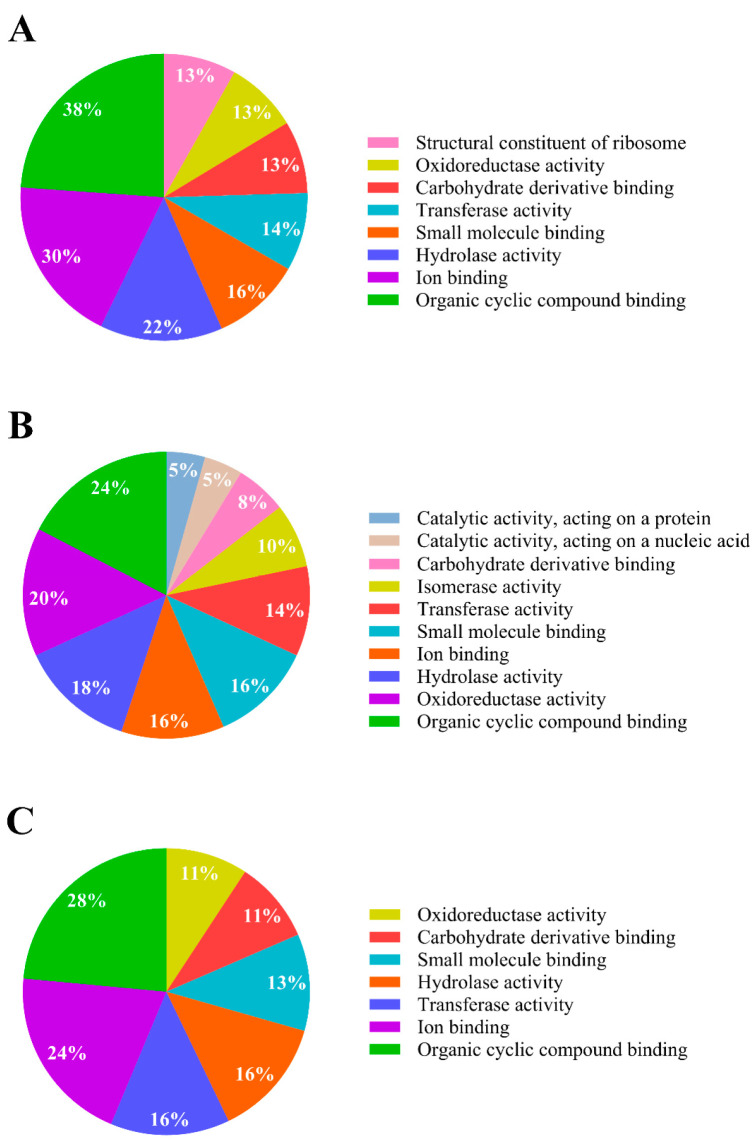
Molecular functional activity analysis of *Pseudomonas* sp. C9 in response to IPR. (**A**) Upregulated proteins; (**B**) downregulated proteins; (**C**) proteins expressed only in IPR treatment. Modified from OmicsBox software version 3.1 [15].

**Figure 4:**
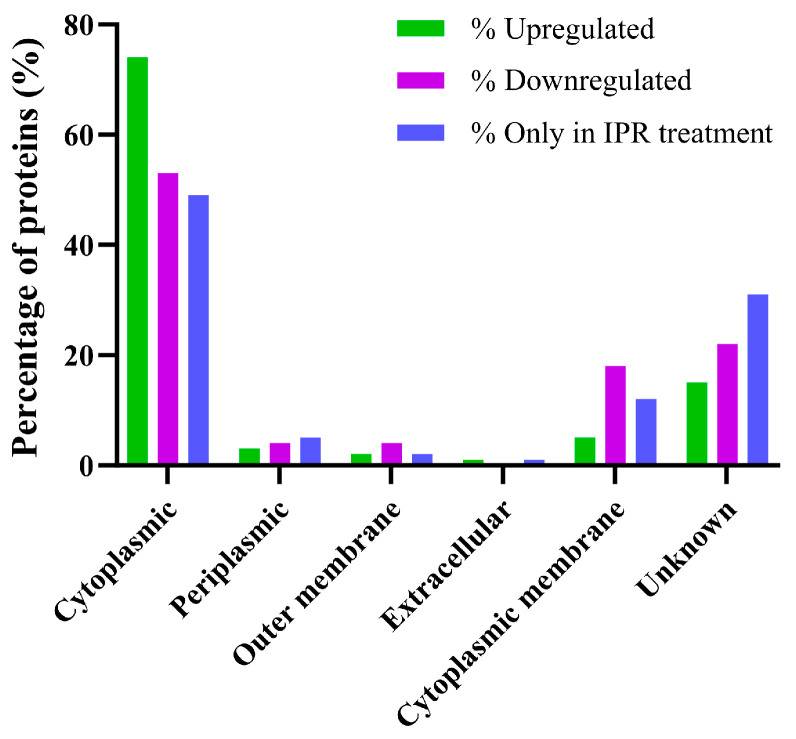
Cellular localisation of proteins that are differentially regulated in response to iprodione in *Pseudomonas* sp. C9.

Functional analysis using OmicsBox software version 3.1 with the NCBI database showed that 60% of the upregulated proteins possess catalytic activities, and 51% possess binding activities. Of those proteins with catalytic activities, 22% have hydrolase activities, 14% transferase, and 13% oxidoreductase (Figure 3A). Of those with binding activities, 38% have organic cyclic compound binding, 30% ion binding, 16% small molecule binding, and 13% carbohydrate derivative binding. These proteins were located primarily in the cytoplasm of the cell, representing 74% of the identified proteins (Figure 4).

The significantly upregulated proteins analysed using the KEGG^®^ database identified 76 pathways that might aid the organism in the degradation and tolerance of the pesticide IPR (Table S2). The proteins in these upregulated pathways are mainly involved in the biosynthesis of secondary metabolites, ribosomes, microbial metabolism in diverse environments, carbon metabolism, and biosynthesis of amino acids.

Among the upregulated proteins, the list of those significantly upregulated is provided in Table S1. In Table 1, we identified the upregulated proteins with a fold change ≥4.0, including two catalase enzymes [EC 1.11.1.6], peptidoglycan-binding protein LysM, guanylate kinase [EC:2.7.4.8], acetyl-CoA carboxylase biotin carboxyl carrier protein, Type 1 glutamine amidotransferase, 2,3,4,5-tetrahydropyridine-2,6-dicarboxylate N-succinyltransferase [EC:2.3.1.117], 30S ribosomal protein S6, Electron transfer flavoprotein subunit beta/FixA family protein, Grx4 family monothiol glutaredoxin, OsmC family protein, and efflux resistance–nodulation–division (RND) transporter periplasmic adaptor. These proteins are expressed in response to the stress caused by IPR. Additionally, among the upregulated proteins, we identified those involved in chemical degradation, such as formaldehyde dehydrogenase (fdhA), dhcA, 3-oxo adipate enol-lactonase, and ddlB. These proteins are involved in xenobiotic degradation and could be associated with IPR degradation. Among the hydrolase enzymes, we found an amidohydrolase [EC:3.5.1.-] which is likely involved in IPR degradation (Table 1).

### 2.4. Downregulated Proteins

For downregulated proteins, the significantly expressed ones were analysed according to their molecular function. We found that 63% of the proteins possess catalytic activities, while 27% have binding activities. Of the catalytic activities, 20% are oxidoreductases, 18% are hydrolases, and 14% are transferases, among others (Figure 3B). Additionally, we found that binding activities were present. Among these, 24% of the proteins bind to organic cyclic compounds, and 16% bind to small molecules and ions, respectively. These proteins were primarily located in the cytoplasm of the cell (Figure 4).

The proteins were also analysed using the KEGG^®^ database, identifying 32 metabolic pathways affected by IPR treatment (Table S2). The proteins most affected were involved in the following metabolic pathways: biosynthesis of secondary metabolites, biosynthesis of cofactors, glutathione metabolism, bacterial secretion system, butanoate metabolism, biosynthesis of nucleotide sugars, oxidative phosphorylation, two-component system, and biofilm formation.

Of the 51 downregulated proteins, the most affected protein, maleylacetoacetate isomerase [EC:5.2.1.2], has a fold change of ≤−2.0 and participates in the styrene degradation metabolic pathway with catalytic activity. Among the downregulated proteins, we found those that act on DNA damage, such as class II aldolase/adducin family protein and cold-shock protein with actin filament binding and nucleic acid binding activities, respectively. Furthermore, a variety of proteins possess oxidoreductase and hydrolase activities.

### 2.5. Proteins Expressed Only in IPR Treatment

The 382 proteins expressed only in response to IPR treatment were analysed for their molecular function. We found that 47% of these proteins possess catalytic activity, with 11% having oxidoreductase activities, 16% having transferase activities, and 16% having hydrolase activities. Additionally, 39% possess binding activities, with 28% of these proteins binding to organic cyclic compounds (Figure 3C), including nucleic acids (DNA and RNA) and nucleotides. Like the up- and downregulated proteins, they are mainly located in the cytoplasm (Figure 4).

According to the KEGG^®^ database, these proteins participate in 81 metabolic pathways, principally in the biosynthesis of secondary metabolites, two-component systems, microbial metabolism in diverse environments, biosynthesis of amino acids, and biosynthesis of cofactors (Table S2).

## 3. Discussion

Soil bacteria are microorganisms capable of degrading pesticides through enzymatic reactions, thereby potentially reducing their environmental impact. Among soil bacteria, the genus *Pseudomonas* has been reported to degrade various pesticides, including endosulfan, chlorpyrifos, diuron, atrazine, and IPR [7,21,22,23]. In this study, an IPR concentration of 50 mg L^−1^ and an exposure time of 24 h were used, as previous studies have shown that the C9 strain requires nearly 10 h to degrade 50% of the pesticide, with this percentage increasing to 80% at 24 h [7,13]. Therefore, the entire enzymatic machinery should be actively engaged in the degradation of the pesticide during this period. Transformation of 50 mg L^−1^ IPR to *N*-(3,5-dichlorophenyl)-2,4-dioxoimidazolidine (II) and under restrictive conditions to 3,5-dichlorophenylurea acetic acid (III) was reported for *Pseudomonas fluorescens, Pseudomonas* sp., while *Pseudomonas paucimobilis* was responsible for degrading II to III and III to 3,5-dichloroaniline [11].

The degradation of pesticides provides these bacteria with a new source of carbon and can occur through hydrolytic pathways, as observed by Zaffar et al. [24]. In this study, we identified 1472 proteins that were differentially expressed in response to IPR exposure in the strain *Pseudomonas* sp. C9. Similar to Aswathi et al. [25], where proteomic analysis of *Pseudomonas nitroreducens* revealed 1316 proteins in response to chlorpyrifos pesticide exposure, our findings suggest that exposure to IPR triggers significant changes in the protein expression of *Pseudomonas* sp. C9. These changes in protein expression may be associated with the enzymatic pathways involved in the degradation of IPR or other adaptive responses to tolerate the pesticide.

It was previously shown that exposure to IPR significantly influences metabolic and cellular processes, as observed in *Achromobacter* sp. C1 in response to IPR treatment [13]. This influence is also reflected in their catabolic activities, where transferase and hydrolase activities are the most affected in strain C9. Overall, the proteomic analysis of *Pseudomonas* sp. C9 suggests that exposure to IPR can cause notable alterations in bacterial metabolic and cellular processes, potentially leading to changes in enzymatic pathways involved in pesticide degradation. Furthermore, as observed in the studies by Aswathi et al. [25] and Donoso-Piñol et al. [13], our research on *Pseudomonas* sp. C9 showed that among upregulated proteins, the most affected metabolic pathways are the biosynthesis of secondary metabolites and microbial metabolism in diverse environments (Table S1). Moreover, among the upregulated proteins, the most significant catalytic activities are hydrolase and transferase activities, which are linked to detoxification, molecular transport, modification and maintenance of the bacterial cell wall, cell viability and growth, and degradation of aromatic compounds.

**Figure 5:**
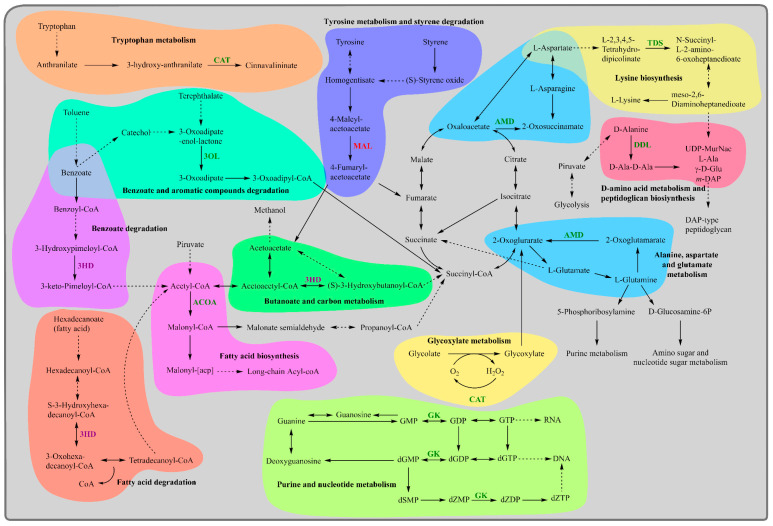
Scheme representing the metabolic changes caused by iprodione exposure in the strain *Pseudomonas* sp. C9. In green letters upregulated proteins, in red letters downregulated proteins, and in purple letters proteins solely expressed on IPR exposure are represented. CAT, catalase; GK, guanylate kinase; TDS, 2,3,4,5-tetrahydropyridine-2,6-dicarboxylate-N-succinyltransferase; ACOA, acetyl-CoA carboxylate biotin carboxyl carrier protein; DDL, D-alanine–D-alanine ligase; (3OL) 3-oxiadipate enol-lactonase; AMD, amidohydrolase; 3HD, 3-hydroxyacyl-CoA dehydrogenase; MAL, maleyacetoacetate isomerase.

For detoxification, we identified catalase as the most upregulated enzyme [EC:1.11.1.6], which is involved in the detoxification of reactive oxygen species (ROS) through the decomposition of hydrogen peroxide to release oxygen, a part of tryptophan and glyoxylate metabolism (Figure 5). Bacterial strains such as *Escherichia coli* K12, *Bacillus subtilis* B19, and *Pseudomonas* sp. CMA 6.9 have demonstrated increased catalase activity when exposed to atrazine or the herbicide Heat^®^ (active ingredient saflufenacil), respectively, indicating a response to oxidative stress [26,27]. The upregulation of catalase activity in strain C9 due to IPR treatment suggests that the strain is employing a similar adaptive response to oxidative stress caused by IPR, enhancing strain C9 capability to neutralise ROS. Additionally, among the upregulated proteins associated with detoxification, we identified guanylate kinase [EC:2.7.4.8], an enzyme involved in nucleotide metabolism for DNA and RNA synthesis, which reduces errors in RNA synthesis during oxidative stress [28]. The OsmC family protein, which is involved in the response to osmotic stress via peroxiredoxin function, reduces peroxide generated during stress and removes ROS [29,30]. Additionally, the upregulated protein Grx4 is a monothiol glutaredoxin, which is part of purine and nucleotide metabolism (Figure 5).

Previously, in *Pseudomonas aeruginosa*, GrxD, a monothiol glutaredoxin, was found to be essential for oxidative stress protection, acting as an electron donor for the organic hydroperoxide resistance enzyme during cumene hydroperoxide degradation [31]. The differential expression of catalase, guanylate kinase, OsmC family proteins, and Grx4 in *Pseudomonas* sp. C9 exposed to IPR suggests that the strain employs a range of adaptive responses to combat oxidative stress and enhance its ability to detoxify ROS. For molecular transport, efflux RND transporter periplasmic adaptors, such as AcrA and AcrE, play a role in the assembly and function of RND efflux pumps in bacteria and contribute to antibiotic resistance by exporting various classes of antibiotics out of the bacterial cell [32]. For strain C9, the differential expression of these proteins indicates that upregulated efflux pumps actively export IPR and other potentially toxic compounds out of the cell, enabling the strain to enhance its resistance to the pesticide.

Among the significantly upregulated proteins associated with the modification and maintenance of the bacterial cell wall, the peptidoglycan-binding protein LysM helps bacteria retain proteins within their cell envelopes by attaching to peptidoglycan, the primary component of bacterial cell walls [33], and glutamine amidotransferase catalyses the transfer of amide nitrogen from glutamine to specific acceptor substrates and is involved in the modification of cell wall peptidoglycan [34]. Moreover, D-alanine–D-alanine ligase (Ddl) [EC:6.3.2.4] is an enzyme involved in the biosynthesis of peptidoglycan and D-amino acid metabolism (Figure 5). Inhibition of Ddl activity has been explored as a potential strategy for developing novel antibacterial agents [35,36,37]. Furthermore, 2,3,4,5-tetrahydropyridine-2,6-dicarboxylate N-succinyltransferase [EC:2.3.1.117], also known as tetrahydrodipicolinate N-succinyltransferase, is involved in the biosynthesis of L-lysine (Figure 5), a component of peptidoglycan [38]. The differential expression of these proteins in strain C9 suggests they potentially contribute to the strain’s ability to withstand the toxic effects of IPR treatment. In terms of cell viability and growth, the upregulation of acetyl-CoA carboxylase biotin carboxyl carrier protein indicates a heightened requirement for fatty acid synthesis [39] (Figure 5). This is associated with cellular damage, as fatty acids are vital components of bacterial cell membranes and require significant energy for production, making their regulation crucial for bacterial viability [40]. This finding aligns with what was observed by Donoso-Piñol et al. [13], where *Pseudomonas* sp. C9 was exposed to IPR, and the biomass was duplicated in comparison to the control condition. Additionally, ribosomal protein S6, a component of the 30S ribosomal subunit in bacteria, plays a role in ribosome assembly and is involved in the binding of other ribosomal proteins and RNA molecules [41]. Electron transfer flavoprotein (ETF) also plays a crucial role in electron bifurcation and energy conservation in bacteria [42,43]. Depletion of ETF in *Burkholderia cenocepacia* leads to a loss of redox potential and cell viability [44]. In the context of strain *Pseudomonas* sp. C9 exposed to IPR, these proteins are essential for the sustainability of the bacterial cell.

Regarding the degradation of aromatic compounds, 3-oxo adipate enol-lactonase [EC:3.1.1.24] is an enzyme involved in the bioprocessing of lactones [45] and is part of the benzoate and aromatic compound degradation pathways (Figure 5). It also participates in the biodegradation of methyl aromatics in *Pseudomonas reinekei* MT1 [46]. Amidohydrolases [EC:3.5.1.-], also known for their activities as amidase enzymes, play a role in the degradation of pesticides and the detoxification of pesticide residues and are part of the alanine, aspartate, and glutamate metabolism pathways in *Pseudomonas* (Figure 5). A broad-spectrum amidohydrolase gene has been identified in bacteria that can completely degrade substituted urea herbicides within 24 h [47]. In *Pseudomonas*, the amidohydrolase AtzH is suspected to be an enzyme that converts 1,3-dicarboxyurea to allophanate in cyanuric acid catabolism, a common metabolic intermediate in the catabolism of s-triazine compounds, including atrazine and other herbicides [48]. Additionally, the bacterial strain *Ochrobactrum* sp. PP-2, with a unique arylamidase enzyme, effectively transforms the herbicide propanil into a less harmful compound, showing high efficiency and specificity [49]. Overall, our findings indicate that exposure to IPR leads to changes in proteomic expression, suggesting that strain *Pseudomonas* sp. C9 has developed adaptive mechanisms to efficiently degrade IPR and mitigate its potentially harmful effects on the environment.

Among the downregulated proteins, which are proteins whose expression levels decrease in response to a particular condition or treatment, in *Pseudomonas* sp. C9 exposed to IPR, those associated with the degradation of aromatic compounds, glycolysis and gluconeogenesis, and stress adaptations were identified. For the degradation of aromatic compounds such as amino acids, we report maleylacetoacetate isomerase [EC:5.2.1.2], an enzyme involved in the metabolic degradation of phenylalanine and tyrosine (Figure 5) [50]. This decrease could be impacting the ability of the C9 strain to metabolise aromatic compounds effectively.

Additionally, class II aldolases [EC:1.1.2.13] are enzymes that catalyse the reversible cleavage of fructose 1,6-bisphosphate. These zinc-containing metalloproteins play a crucial role in glycolysis and gluconeogenesis, converting three-carbon molecules into six-carbon sugars, and vice versa, thereby supporting energy production and carbon metabolism; in addition, they are involved in the degradation of the hydrocarbon tetralin [51].

Furthermore, we report cold-shock proteins (CS), a group of proteins found in bacteria that are important for cold adaptation and survival. CS proteins are essential for bacterial growth under unfavourable conditions [52,53]. In *Bacillus subtilis*, the loss of CS proteins affects the expression of about 20% of all genes and can lead to growth defects and loss of genetic competence [54].

**Figure 6:**
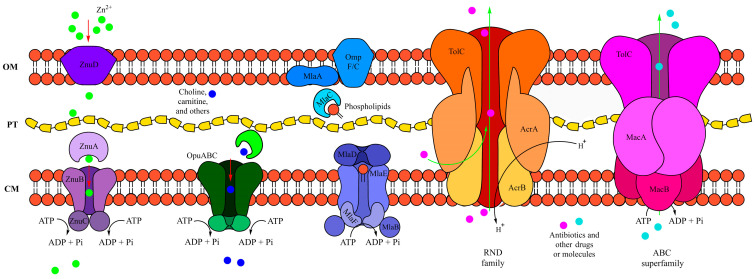
Schematic representation of transporters in *Pseudomonas* sp. C9. From left to right: Znu ABC import system, osmoprotectant uptake (Opu) system, Mla ABC transport system, RND family TolC AcrAB system, and ABC superfamily TolC MacAB outer membrane protein system [16,17,18,19,20].

Overall, our findings suggest that exposure to IPR inhibits key proteins. However, we also observed that the strain *Pseudomonas* sp. C9 has developed adaptive mechanisms to efficiently degrade IPR and mitigate its potentially harmful effects through upregulated proteins and those proteins that are only present with IPR treatment. Moreover, among the proteins only present with IPR treatment, we found those associated with transport, multidrug efflux complexes, chemical degradation, β-lactam resistance, and CAMP resistance. For transport, we identified ABC transporters; these proteins are primary active transporters that move substrates across biological membranes using ATP as an energy source (Figure 6).

In bacteria, they are involved in processes such as drug resistance (Mac system), LPS transport (Lpt system), phospholipid transport (Mla system), and lipoprotein transport to the outer membrane (Lol system). For instance, the Mac system is an ABC transporter-based nanomachinery that contributes to bacterial drug resistance and is part of antibiotic resistance mechanisms [55,56]. In strain C9, we identified six proteins belonging to ABC transporters. Among them, the protein ZnuA (Figure 6) is an essential component for transporting zinc across the cell membrane, with ZnuB assisting in zinc uptake. This transport system is crucial for bacterial survival in zinc-limiting environments and is involved in various cellular processes, including virulence [57]. Additionally, we report a choline ABC transporter substrate-binding protein, such as Opu-family proteins, which have been studied in *Bacillus subtilis* and are part of the ABC transporters responsible for acquiring compatible solutes under osmotic stress [58]. The Mla system (Figure 6) is involved in maintaining lipid asymmetry in the outer membrane of Gram-negative bacteria and includes proteins such as MlaA and MlaD [16,59]. The Mla system is also implicated in antibiotic resistance in certain pathogens, including *Acinetobacter baumannii* and *Pseudomonas aeruginosa* [60]. Additionally, the AdeC/AdeK/OprM family belongs to a multidrug efflux complex associated with β-lactamase resistance (Figure 6). These proteins are involved in the transport of various antibiotics and xenobiotics in Gram-negative bacteria. For example, the AdeABC efflux pump in *Acinetobacter baumannii* is a notable RND efflux system that expels antibiotics, similar to the outer membrane factor (OprM) in *Pseudomonas aeruginosa*, leading to multidrug resistance [17,61]. For chemical degradation, 3-hydroxyacyl-CoA dehydrogenase is part of the benzoate degradation pathway, where benzoate, a common aromatic compound found in various environmental sources, is converted to acetyl-CoA via several enzymatic steps. This enzyme was reported to be significantly upregulated in *Enterobacter* sp. Z1 during the degradation of triazophos, methamidophos, and carbofuran pesticides [62]. In our results, this protein was found only when exposed to IPR. Additionally, we report upregulated proteins related to CAMP resistance, similar to those observed in a *Pseudomonas* strain exposed to chlorpyrifos by Aswathi et al. [25]. These proteins may assist *Pseudomonas* strains in pesticide degradation and resistance.

## 4. Materials and Methods

### 4.1. Chemicals and Medium

An analytical standard of IPR with a purity of 99% was procured from Sigma-Aldrich (St. Louis, MO, USA). For the pesticide exposure assay, a formulated commercial IPR (Rovral 50 WP) was obtained from Agan Chemical Manufacturers Ltd. (Ashdod, Israel). A stock solution containing 10,000 mg L^−1^ of IPR was prepared by dissolving the pesticide in dimethyl sulfoxide, then filtering it through a 0.22 µm polytetrafluoroethylene filter before storing it at 4 °C until required. All other chemicals and solvents used were of analytical reagent grade (Merck-Sigma, St. Louis, MO, USA).

The microbiological procedures utilised Luria Bertani modified broth with the following components per litre—2.5 g NaCl, 2.5 g yeast extract, and 5.0 g tryptone—adjusted to pH 6.5. The broth was autoclaved at 121 °C.

### 4.2. Proteome Preparation, Digestion, and Mass Spectrometry Analysis

The strain used in this study was *Pseudomonas* sp. C9 (Accession No. MK110046), an IPR-degrading strain that was isolated from an organic biomixture sample obtained from a BPS used for the treatment of pesticide residues, including IPR [7]. The partial sequence of the 16S ribosomal RNA gene of the strain is available in GenBank with the accession number MK110046. This strain has a genome of 6.9 Mb with 6296 protein-coding genes. The strain was exposed to 50 mg L^−1^ of IPR for 24 h, after which the biomass was collected by centrifugation at 8000× *g* for 20 min at 4 °C and rinsed twice with phosphate-buffered saline (PBS) adjusted to pH 7.0. Following centrifugation, the biomass pellet was suspended in 5 mL of PBS buffer with the addition of a protease inhibitor solution at a concentration of 1 mM. Subsequently, the cells were disrupted using ultrasonication with an amplitude of 40% in 8 cycles of 30 s on ice and subjected to another round of centrifugation at 8000× *g* for 15 min at 4 °C. The resulting supernatant was collected and lyophilised to preserve its integrity [13,63].

For desalting, the sample was suspended in 200 μL of MilliQ© water (Merck, Darmstadt, Germany) and 800 μL of cold acetone, then stored at −30 °C. The sample underwent centrifugation twice at 10,000× *g* for 30 min at 4 °C; the supernatant was discarded, and the extracts were air-dried. The protein extracts were reconstituted with a mix of 500 μL ammonium bicarbonate (25 mM) and 250 μL of MilliQ© water before the protein concentration was verified using the Qubit™ 2.0 Fluorometer (Invitrogen, Carlsbad, CA, USA) according to the manufacturer’s instructions. The protein concentration was then established at 100 µg. An aliquot of dithiothreitol was added to the sample to obtain a final concentration of 10 mM. Next, the samples were homogenised and incubated at 800 rpm and 60 °C for 1 h. After incubation, iodoacetamide was added to obtain a final concentration of 40 mM. Samples were then incubated for 30 min in the dark [13].

Trypsin digestion was performed using a 1:100 ratio of protein to 50 mM ammonium bicarbonate buffer. The sample was treated with 75 μL of trypsin (equivalent to 0.02 µg) and incubated at 800 rpm and 37 °C for 20 h. The trypsinisation process was stopped by adding trifluoroacetic acid (TFA, 1%) to lower the pH value. Subsequently, stage tip columns made with Applied Biosystems™ POROS™ R2 resin (Thermo Fisher Scientific, Waltham, MA, USA) were used for clean-up; elution was carried out using an ACN gradient (50% and 70%). Finally, the peptides were dried at 45 °C for 1 h using a SpeedVac concentrator (Thermo Fisher Scientific, Waltham, MA, USA), then suspended in 100 µL of 0.1% formic acid following standard procedures before quantification with the Qubit™ 2.0 Fluorometer protein assay kit [64].

The samples were analysed in technical triplicates using nano-liquid chromatography (Easy-nLC 1000, Thermo Fisher Scientific, Waltham, MA, USA) coupled to a hybrid quadrupole Orbitrap mass spectrometer (Q Exactive Plus, Thermo Fisher Scientific, Waltham, MA, USA). Peptides from the samples were loaded onto a home-made C18 trap column (Dr. Maisch GmbH, Ammerbuch, Germany) and then separated using a home-made C18 New Objective PicoFRIT column (Dr. Maisch GmbH, Ammerbuch, Germany). The chromatographic flow rate was 0.3 mL min^™1^ with the application of a linear gradient starting at 100% mobile phase A and increasing to 40% mobile phase B over 180 min. Ionisation and transfer of peptides occurred via a nanoelectrospray source with positive polarity, at a potential of 3.0 kV, and heating at 250 °C [13].

The mass spectrometer was operated in data-dependent analysis mode with dynamic exclusion of 45 ms and full-scan MS1 spectra with a resolution of 70,000 at *m*/*z* 200. This was followed by fragmentation of the top 15 most intense ions using high-collision dissociation (HCD), a normalised collision energy (NCE) of 30, and a resolution of 17,500 at *m*/*z* Acce200 in MS/MS scans. Additionally, species with a charge of +1 or greater than +4 were excluded from the MS/MS analysis.

### 4.3. Proteomic Data Analysis

The mass spectrometric data were analysed using Proteome Discoverer 2.1 software. Peptide identification was carried out with the Sequest HT algorithm against a protein database, including common contaminants in mass spectrometry-based proteomics analysis, concatenated with the *Pseudomonas* (Taxon ID 76760) database available from UniProt at http://www.uniprot.org/ (accessed on 15 November 2019). The searches utilised parameters such as peptide mass tolerance, MS/MS range, trypsin cleavage, missed cleavage limit, and fixed and variable modifications. False discovery rates were obtained, and protein quantification was performed using an extracted ion chromatogram (XIC) via the Precursor Ions Area Detector tool. The top 3 methods were used for protein quantification, selecting peptides that were considered unique [13].

### 4.4. Pathway Analysis

Identified proteins were then analysed using BLAST2Go software version 4.1 [14] and OmicsBox software version (3.1.11) [15] with the NCBI databank [65] to identify their molecular functions. To map the proteins in various possible pathways, the KEGG© database [66] was used to visualise the proteins in the metabolic pathway of *Pseudomonas* sp. C9 under IPR exposure, using KEGG© Mapper to visualise the upregulated and downregulated proteins, and proteins present only in the presence of IPR. To determine the cellular location, we used PSORTb version 3.0 [67] for subcellular localisation prediction.

### 4.5. Statistical Analysis

All experiments were performed in triplicate, and the standard deviation was calculated for protein expression. The ratio between quantitative values from IPR exposure (B) and control (A) was determined in triplicate for each protein, and the mean was used to calculate the fold change (FC, B/A). To normalise the data, the fold change value was calculated with log2. To determine the significant values, the log2FC—median was calculated, and values within the [−SD–+SD] range of the median standard deviation were considered significant.

## 5. Conclusions

In conclusion, the BPS bacterium *Pseudomonas* sp. C9, an IPR-degrading strain, was studied through comparative proteomics, revealing several differentially expressed proteins and proteins expressed only under IPR treatment. These proteins may play roles in IPR tolerance and degradation and are primarily involved in metabolic and cellular processes, with predominant molecular functions related to transferase and hydrolase activities. The upregulation of certain proteins and the presence of proteins unique to IPR treatment indicate the bacterium’s capacity to adapt and respond to the pesticide, demonstrating its ability to degrade and detoxify IPR. Additionally, these proteins are involved in ROS detoxification, growth, and transport, which may enhance the C9 strain’s resistance to IPR exposure. These results suggest that *Pseudomonas* sp. C9 has developed adaptive strategies for effective IPR degradation and tolerance, despite the downregulation of certain proteins. The BPS provides a valuable source of adapted microorganisms that can be used in bioremediation strategies. Finally, proteomic analysis of pesticide-degrading bacteria is essential for identifying key proteins involved in the breakdown and detoxification of these compounds. This technique reveals how the bacterium adapts its metabolism and responds to stress caused by pesticides. These findings provide crucial insights for improving bioremediation strategies, as they inform the development of microbial strains with enhanced environmental detoxification capacity.

## Figures and Tables

**Table 1:** *Pseudomonas* sp. C9 identified proteins differentially expressed under 50 mg L^−1^ IPR exposure.

Accession	Protein Name	FC	Function
**Upregulated proteins**			
A0A5C5N9R8	Catalase	8.6	Heme binding, catalase activity
A0A5C5NYY2	Peptidoglycan-binding protein LysM	7.2	Involved in binding peptidoglycan in bacteria
A0A5C5NZ78	Guanylate kinase	5.6	Guanylate kinase activity
A0A5C5NIP7	Catalase HPII	5.5	Heme binding, catalase activity
A0A5C5NJ26	Type 1 glutamine amidotransferase	5.3	Glutamine amidotransferase activity
A0A5C5P0X8	Acetyl-CoA carboxylase biotin carboxyl carrier protein	5.0	Acetyl-CoA carboxylase activity
A0A5C5NX76	2,3,4,5-tetrahydropyridine-2,6-dicarboxylate N-succinyltransferase	4.9	2,3,4,5-tetrahydropyridine-2,6-dicarboxylate N-succinyltransferase activity
A0A5C5NZ12	30S ribosomal protein S6	4.6	Structural constituent of ribosome, rRNA binding
A0A5C5NV16	Electron transfer flavoprotein subunit beta/FixA family protein	4.5	Electron transfer activity
**Accession**	**Protein Name**	**FC**	**Function**
A0A5C5NXV6	Grx4 family monothiol glutaredoxin	4.5	Disulfide oxidoreductase activity
A0A5C5NT48	OsmC family protein	4.3	Stress-induced protein
A0A5C5NZ11	Adenosylhomocysteinase	4.2	Adenosylhomocysteinase activity
A0A5C5NV61	Efflux RND transporter periplasmic adaptor	4.1	Transmembrane transporter activity
A0A5C5NCX7	Formaldehyde dehydrogenase, glutathione-independent fdhA	2.6	Oxidoreductase activity, zinc ion binding
A0A5C5N9R9	Dehydrocarnitine CoA-transferase subunit A (dhcA)	1.3	CoA-transferase activity
A0A5C5NV30	3-oxoadipate enol-lactonase	1.7	3-oxoadipate enol-lactonase activity
A0A5C5NPA5	D-alanine–D-alanine ligase (ddlB)	1.8	ATP binding, D-alanine–D-alanine ligase activity, metal ion binding
A0A5C5NT33	Amidohydrolase	2.2	Amidase activity
**Downregulated proteins**			
A0A5C5NF46	Maleylacetoacetate isomerase	−2.0	Catalytic activity
A0A5C5NUW1	Cold-shock protein	−1.7	Nucleic acid binding
**Accession**	**Protein Name**	**FC**	**Function**
A0A5C5NS33	Class II aldolase/adducin family protein	−1.7	Actin filament binding
**Only expressed in** **IPR treatment proteins**			
A0A5C5NPY3	Zinc ABC transporter substrate-binding protein ZnuA	Unique	Metal ion binding
A0A5C5NXG4	Choline ABC transporter substrate-binding protein	Unique	Choline binding, transmembrane transporter activity
A0A5C5NMV1	Outer membrane lipid asymmetry maintenance protein MlaD	Unique	Phospholipid transporter activity
A0A5C5NWW8	AdeC/AdeK/OprM family multidrug efflux complex outer membrane factor	Unique	Transmembrane transporter activity, efflux transmembrane transporter activity
A0A5C5NCA5	Efflux RND transporter periplasmic adaptor subunit	Unique	Transmembrane transporter activity
A0A5C5NJP5	Beta-lactamase	Unique	β-lactamase activity
A0A5C5NVP6	3-hydroxyacyl-CoA dehydrogenase	Unique	Catalytic activity, oxidoreductase activity
A0A5C5N839	Vanillate O-demethylase oxidoreductase VanB	Unique	Undetermined
A0A5C5N7Z3	CoA-transferase subunit B	Unique	CoA-transferase activity

Source: [14,15,16,17,18,19,20].

## Data Availability

Data is contained within the article and supplementary material.

## Supplementary Materials

The following supporting information can be downloaded at https://www.mdpi.com/article/10.3390/ijms251910471/s1.
