# Supplementary material for: Proteome Changes Induced by Iprodione Exposure in the Pesticide-Tolerant Pseudomonas sp. C9 Strain Isolated from a Biopurification System
Source: Int J Mol Sci. 2024 Sep 28;25(19):10471. doi: 10.3390/ijms251910471 (PMC11476656; doi:10.3390/ijms251910471)
Supplement: Supplementary file 1 [file ijms-25-10471-s001.zip › ijms-3221679-supplementary.pdf]

Supplementary Table S1. Identified proteins for *Pseudomonas sp. C9*.

Upregulated proteins			Downregulated proteins			Proteins only in IPR treatment		
**Accession**	**Protein name**	**FC**	**Accession**	**Protein name**	**FC**	**Accession**	**Protein name**	**FC**
A0A5C5N9R8	Catalase	8.6	A0A5C5NZ64	Maleylacetoacetate isomerase	-2.0	A0A5C5NUQ3	Maltose alpha-D-glucosyltransferase	U
A0A5C5NYY2	Peptidoglycan-binding protein LysM	7.2	A0A5C5NHU5	DUF4426 domain-containing protein	-1.8	A0A5C5NC19	DNA ligase D	U
A0A5C5NZ78	Guanylate kinase	5.6	A0A5C5NFE0	Cold-shock protein	-1.7	A0A5C5NZM0	Dimethylglycine demethylation protein DgcA	U
A0A5C5NIP7	Catalase HPII	5.5	A0A5C5NGA5	Class II aldolase/adducin family protein	-1.7	A0A5C5NGL5	Transketolase	U

A0A5C5NJ26	Type 1 glutamine amidotransferase	5.3	A0A5C5NGL0	Re/Si-specific NAD(P)(+) transhydrogenase subunit alpha	-1.7	A0A5C5NG27	BON domain-containing protein	U
A0A5C5P0X8	Acetyl-CoA carboxylase biotin carboxyl carrier protein	5.0	A0A5C5NZ71	PasA protein	-1.7	A0A5C5NQD8	Ubiquinone-dependent pyruvate dehydrogenase	U
A0A5C5NZ12	30S ribosomal protein S6	4.6	A0A5C5P1H8	Enoyl-CoA hydratase/isomerase family protein	-1.5	A0A5C5NXB6	DEAD/DEAH box helicase	U
A0A5C5NV16	Electron transfer flavoprotein subunit beta/FixA family protein	4.5	A0A5C5NYT5	Leucyl aminopeptidase	-1.5	A0A5C5NN96	PQQ-dependent dehydrogenase. methanol/ethanol family	U

A0A5C5NXV6	Grx4 family monothiol glutaredoxin	4.5	A0A5C5NB23	Cation acetate symporter	-1.5	A0A5C5NT29	N-acetylglutaminylglutamine amidotransferase	U
A0A5C5NT48	OsmC family protein	4.3	A0A5C5NMT5	Cobalamin biosynthesis protein CobW	-1.5	A0A5C5NA49	Lactonase family protein	U
A0A5C5NZ11	Adenosylhomocysteinase	4.2	A0A5C5NM57	5-(Carboxyamino)imidazole ribonucleotide mutase	-1.5	A0A5C5NKS1	DUF4142 domain-containing protein	U
A0A5C5NV61	Efflux RND transporter periplasmic adaptor subunit	4.1	A0A5C5NJH9	SPFH/Band 7/PHB domain protein	-1.5	A0A5C5NRX3	DUF3416 domain-containing protein	U

A0A5C5NX16	HAD family hydrolase	3.7	A0A5C5NKL9	Aminodeoxychorismate/anthranilate synthase component II	-1.5	A0A5C5P0U2	Bacterioferritin	U
A0A5C5NP21	Elongation factor P	3.5	A0A5C5P0E9	30S ribosome-binding factor RbfA	-1.5	A0A5C5NV70	Dihydroorotase	U
A0A5C5NPV2	Dipeptidase	3.4	A0A5C5NZ14	Polyphosphate kinase 1	-1.5	A0A5C5NK38	3-isopropylmalate dehydratase large subunit	U
A0A5C5NS15	YajQ family cyclic di-GMP-binding protein	3.4	A0A5C5NWC9	Amidotransferase	-1.4	A0A5C5NLU4	PAS domain-containing protein	U
A0A5C5N9B2	30S ribosomal protein S4	3.4	A0A5C5NH08	Metal-binding protein	-1.4	A0A5C5NZY1	Nucleoid-associated protein YejK	U
A0A5C5N9B3	50S ribosomal protein L3	3.4	A0A5C5NW54	Uncharacterized protein	-1.4	A0A5C5NIK5	Pilus assembly protein PilZ	U

A0A5C5P048	Imidazoleglycerol-phosphate dehydratase HisB	3.3	A0A5C5NDP5	2-hydroxy-3-oxopropionate reductase	-1.4	A0A5C5NJ53	Hemerythrin domain-containing protein	U
A0A5C5NH99	Sigma-54-dependent Fis family transcriptional regulator	3.3	A0A5C5NAX5	23S rRNA (Uridine(2552)-2'-O)-methyltransferase RlmE	-1.4	A0A5C5NEL0	Xanthine dehydrogenase family protein subunit M	U
A0A5C5N7Y6	Carboxymuconolactone decarboxylase family protein	3.2	A0A5C5NX61	Fumarate/nitrate reduction transcriptional regulator Fnr	-1.4	A0A5C5NVH8	NAD-dependent DNA ligase LigA	U
A0A5C5NE62	Inorganic diphosphatase	3.2	A0A5C5NNN9	Porin	-1.4	A0A5C5NRK9	Methionine synthase	U
A0A5C5N9G8	50S ribosomal protein L10	3.2	A0A5C5NUS8	Glutathione S-transferase family protein	-1.4	A0A5C5NDB3	50S ribosomal protein L18	U

A0A5C5NTC8	Co-chaperone GroES	3.2	A0A5C5NK22	FAD-binding oxidoreductase	-1.4	A0A5C5NAT1	YeaH/YhbH family protein	U
A0A5C5NEL4	Glycine-betaine demethylase subunit GbcA	3.2	A0A5C5NX15	Cytochrome-c oxidase. cbb3-type subunit III	-1.4	A0A5C5NX66	DUF4197 domain-containing protein	U
A0A5C5NEZ3	F0F1 ATP synthase subunit epsilon	3.1	A0A5C5NTK5	Methyl-accepting chemotaxis protein	-1.4	A0A5C5NUW8	Putative 4-hydroxy-4-methyl-2-oxoglutarate aldolase	U
A0A5C5NES7	Isocitrate lyase	3.0	A0A5C5NTF7	Type VI secretion system membrane subunit TssM	-1.4	A0A5C5NB29	50S ribosomal protein L23	U
A0A5C5NT66	Holliday junction branch migration protein RuvA	2.9	A0A5C5NH68	Molecular chaperone SurA	-1.4	A0A5C5NZC4	S41 family peptidase	U

A0A5C5NFJ4	NADP-dependent isocitrate dehydrogenase	2.9	A0A5C5NP46	UDP-N-acetylmuramate:L-alanyl-gamma-D-glutamyl-meso-diaminopimelate ligase	-1.3	A0A5C5NAL1	LPS-assembly protein LptD	U
A0A5C5NS20	30S ribosomal protein S16	2.8	A0A5C5NTS7	Ribonuclease T	-1.3	A0A5C5NG75	Glutamine-hydrolyzing carbamoyl-phosphate synthase small subunit	U
A0A5C5NVJ0	DUF4398 domain-containing protein	2.8	A0A5C5NFA5	Aldo/keto reductase	-1.3	A0A5C5NX70	Type VI secretion system-associated FHA domain protein TagH	U
A0A5C5N9A0	50S ribosomal protein L14	2.8	A0A5C5NTU7	Thioredoxin-disulfide reductase	-1.3	A0A5C5P0S0	Oxidative damage protection protein	U

A0A5C5NBH4	50S ribosomal protein L25/general stress protein Ctc	2.7	A0A5C5NXR3	Beta-glucosidase BglX	-1.3	A0A5C5NH25	OsmC family protein	U
A0A5C5N9C9	30S ribosomal protein S17	2.7	A0A5C5NFY3	EAL domain-containing protein	-1.3	A0A5C5NX09	DUF4399 domain-containing protein	U
A0A5C5NHV0	Molecular chaperone DnaK	2.7	A0A5C5NCQ1	Excinuclease ABC subunit B	-1.3	A0A5C5NXH7	Amino acid ABC transporter ATP-binding protein	U
A0A5C5N857	Peptidase M4 family protein	2.7	A0A5C5NSZ0	N-acetylmuramic acid 6-phosphate phosphatase MupP	-1.3	A0A5C5N9W5	Uncharacterized protein	U
A0A5C5N900	Glycogen debranching protein GlgX	2.7	A0A5C5NXV3	Nucleotide sugar dehydrogenase	-1.3	A0A5C5NXN6	Cytochrome c oxidase accessory protein CcoG	U

A0A5C5N979	Malto-oligosyltrehalose trehalohydrolase	2.6	A0A5C5NWP8	GNAT family N-acetyltransferase	-1.3	A0A5C5ND72	Peptide chain release factor 1	U
A0A5C5NJE0	Transaldolase	2.6	A0A5C5NQF8	Zn-dependent hydrolase	-1.3	A0A5C5NQ36	TonB-dependent siderophore receptor	U
A0A5C5NRW8	LTXXQ domain protein	2.6	A0A5C5NR82	tRNA uridine-5-carboxymethylaminomethyl(34) synthesis enzyme MnmG	-1.3	A0A5C5P187	Glucose-1-phosphate thymidylyltransferase RfbA	U
A0A5C5NIA3	Peptidylprolyl isomerase	2.6	A0A5C5NRV7	D-amino acid dehydrogenase	-1.3	A0A5C5NPS9	Aminopeptidase P family protein	U
A0A5C5NCX7	Formaldehyde dehydrogenase. glutathione-independent	2.6	A0A5C5NNK3	Class II poly(R)-hydroxyalkanoic acid synthase	-1.3	A0A5C5NFW9	RNA polymerase-binding protein DksA	U

A0A5C5N7S3	Cupin domain-containing protein	2.6	A0A5C5NTM9	Alpha-2-macroglobulin family protein	-1.3	A0A5C5NY47	Cytochrome c5 family protein	U
A0A5C5NB69	F0F1 ATP synthase subunit B	2.6	A0A5C5NLU1	Glycine zipper 2TM domain-containing protein	-1.2	A0A5C5NQU7	DUF883 family protein	U
A0A5C5P119	Branched-chain amino acid ABC transporter substrate-binding protein	2.6	A0A5C5NH92	3-hydroxybutyrate dehydrogenase	-1.2	A0A5C5NCM6	30S ribosomal protein S21	U
A0A5C5NQ11	Type 1 glutamine amidotransferase domain-containing protein	2.6	A0A5C5NUW1	PhzF family phenazine biosynthesis protein	-1.2	A0A5C5NS27	Uncharacterized protein	U
A0A5C5NJ96	30S ribosomal protein S1	2.5	A0A5C5NXG2	Universal stress protein	-1.2	A0A5C5NSA2	D-glycerate dehydrogenase	U

A0A5C5NXY0	Asparaginase	2.5	A0A5C5NF46	YecA family protein	-1.2	A0A5C5NJC1	Class I SAM-dependent methyltransferase	U
A0A5C5N9D7	Single-stranded DNA-binding protein	2.5				A0A5C5N823	DUF4174 domain-containing protein	U
A0A5C5NS74	IMP dehydrogenase	2.5				A0A5C5N9F1	Bacterioferritin	U
A0A5C5NN59	Peptidylprolyl isomerase	2.5				A0A5C5P107	3-dehydroquinate synthase	U
A0A5C5NVS8	NUDIX domain-containing protein	2.5				A0A5C5NE32	Ku protein	U
A0A5C5NMX8	Phosphoheptose isomerase	2.4				A0A5C5NS30	FKBP-type peptidyl-prolyl cis-trans isomerase	U
A0A5C5NY40	Membrane dipeptidase	2.4				A0A5C5NIR5	Uncharacterized protein	U

A0A5C5NDD2	Carbon-nitrogen hydrolase family protein	2.4	A0A5C5NL18	Tryptophan synthase subunit beta	U
A0A5C5NZG7	Bifunctional phosphoribosylaminoimidazolecarboxamide formyltransferase/IMP cyclohydrolase	2.4	A0A5C5N928	Glycogen synthase GlgA	U
A0A5C5NA52	PQQ-dependent sugar dehydrogenase	2.4	A0A5C5NX99	MinD/ParA family protein	U
A0A5C5NN47	YebC/PmpR family DNA-binding transcriptional regulator	2.4	A0A5C5NS47	Glutamate 5-kinase	U

A0A5C5NRJ5	Preprotein translocase subunit SecA	2.3	A0A5C5NHX2	ATP-binding cassette domain-containing protein	U
A0A5C5NDS2	MaoC family dehydratase	2.3	A0A5C5NLZ8	Circadian clock protein KaiC	U
A0A5C5NIY0	Universal stress protein UspA	2.3	A0A5C5NS99	Fe-S protein assembly chaperone HscA	U
A0A5C5NMY4	Ribosome-associated translation inhibitor RaiA	2.3	A0A5C5NVQ4	RNA polymerase sigma factor RpoS	U
A0A5C5NV71	Transcriptional regulator	2.3	A0A5C5NHB0	Transcription elongation factor GreA	U
A0A5C5NTE8	50S ribosomal protein L19	2.3	A0A5C5NJF5	Uncharacterized protein	U

A0A5C5NT33	Amidohydrolase	2.2
A0A5C5NYI1	Class 1 fructose-bisphosphatase	2.2
A0A5C5NPI9	Tail-specific protease	2.2
A0A5C5NPS3	UDP-N-acetylmuramoyl-L-alanyl-D-glutamate--2.6-diaminopimelate ligase	2.2
A0A5C5NXN5	Phosphoglycerate kinase	2.2
A0A5C5NDC2	30S ribosomal protein S3	2.2

A0A5C5NQ34	Cytochrome c-550 PedF	U
A0A5C5NRR5	Bifunctional protein-disulfide isomerase/oxidoreductase DsbC	U
A0A5C5NZF5	Pilin assembly protein	U
A0A5C5NUD3	Glycine zipper 2TM domain-containing protein	U
A0A5C5NNT3	Lipopolysaccharide transport periplasmic protein LptA	U
A0A5C5NN67	DUF1043 family protein	U

A0A5C5NYN4	Type VI secretion system tube protein Hcp	2.2	A0A5C5NIY7	Uncharacterized protein	U
A0A5C5N9C1	30S ribosomal protein S8	2.1	A0A5C5NBY8	CBS domain-containing protein	U
A0A5C5NY02	Electron transfer flavoprotein-ubiquinone oxidoreductase	2.1	A0A5C5NX26	Transcriptional regulator	U
A0A5C5NAY0	Indole-3-glycerol phosphate synthase TrpC	2.1	A0A5C5NX85	YicC family protein	U
A0A5C5NVU2	Formyltetrahydrofolate deformylase	2.1	A0A5C5NXG3	Type VI secretion system protein TssA	U
A0A5C5NUQ4	Peroxiredoxin	2.1	A0A5C5NWH4	Response regulator transcription factor	U

A0A5C5NK80	Uncharacterized protein	2.1	A0A5C5NKU1	Nitrate reductase subunit alpha	U
A0A5C5P055	2.3-bisphosphoglycerate-independent phosphoglycerate mutase	2.1	A0A5C5NUW6	N-acetylglutaminylglutamine synthetase	U
A0A5C5NES3	Topoisomerase II	2.1	A0A5C5NGU1	DUF945 domain-containing protein	U
A0A5C5P081	TldD/PmbA family protein	2.0	A0A5C5NK90	LacI family DNA-binding transcriptional regulator	U
A0A5C5NH60	30S ribosomal protein S15	2.0	A0A5C5NM82	Maltose alpha-D-glucosyltransferase	U
A0A5C5NUE5	Helix-turn-helix domain-containing protein	2.0	A0A5C5NH50	Shikimate dehydrogenase	U

A0A5C5NDI6	Glycine-betaine demethylase subunit GbcB	2.0	A0A5C5NCV3	tRNA (Uridine(54)-C5)-methyltransferase TrmA	U
A0A5C5NM45	Peroxiredoxin	2.0	A0A5C5NF35	OprD family porin	U
A0A5C5NND7	Outer membrane protein assembly factor BamD	2.0	A0A5C5NMX4	Metalloprotease TldD	U
A0A5C5NXD6	Fructose-bisphosphate aldolase class II	2.0	A0A5C5NA85	Helix-turn-helix transcriptional regulator	U
A0A5C5NKA0	Translation initiation factor IF-3	2.0	A0A5C5NXC3	Acetyl-CoA hydrolase/transferase family protein	U
A0A5C5N9B8	50S ribosomal protein L15	1.9	A0A5C5NMX9	Response regulator	U

A0A5C5NXN7	FAD-binding oxidoreductase	1.9	A0A5C5NZD7	Rsd/AlgQ family anti-sigma factor	U
A0A5C5NX05	HU family DNA-binding protein	1.9	A0A5C5NUI1	M48 family metallopeptidase	U
A0A5C5NNU9	Alpha-D-glucose phosphate-specific phosphoglucomutase	1.9	A0A5C5NLB1	Aldo/keto reductase	U
A0A5C5NB10	30S ribosomal protein S13	1.9	A0A5C5NT37	Ankyrin repeat domain-containing protein	U
A0A5C5N9E9	50S ribosomal protein L11	1.9	A0A5C5NYX5	ATP-binding cassette domain-containing protein	U

A0A5C5N8Y8	Peptide-methionine (R)-S-oxide reductase MsrB	1.9	A0A5C5N9V4	ABC transporter substrate-binding protein	U
A0A5C5NIE2	DUF1161 domain-containing protein	1.9	A0A5C5NXN3	DUF4398 domain-containing protein	U
A0A5C5NB64	DNA-directed RNA polymerase subunit alpha	1.9	I1SXX4	Major outer membrane lipoprotein I (Fragment)	U
A0A5C5NHA7	Acetyl-CoA hydrolase/transferase family protein	1.9	A0A5C5NH51	DUF883 domain-containing protein	U
A0A5C5NT78	DUF853 family protein	1.9	A0A5C5NVI5	CsbD family protein	U
A0A5C5P108	Ribonuclease R	1.9	A0A5C5P1I9	Uncharacterized protein	U

A0A5C5NYU7	Outer membrane protein assembly factor BamC	1.9	A0A5C5P1N4	Serine kinase/phosphatase	U
A0A5C5NM09	GNAT family N-acetyltransferase	1.9	A0A5C5NS24	Ubiquinol oxidase subunit II	U
A0A5C5N9G1	50S ribosomal protein L4	1.8	A0A5C5NFW0	Uncharacterized protein	U
A0A5C5NM26	Oxaloacetate decarboxylase	1.8	A0A5C5NEM3	Sarcosine oxidase subunit gamma family protein	U
A0A5C5NBG8	Acyloxyacyl hydrolase	1.8	A0A5C5NF83	Termination factor Rho	U
A0A5C5NRM8	Nitroreductase	1.8	A0A5C5NT44	DUF5064 family protein	U
A0A5C5NDB7	PhoX family phosphatase	1.8	A0A5C5NAW3	GTP cyclohydrolase II	U
A0A5C5NJN7	DUF4404 family protein	1.8	A0A5C5NT52	Hotdog fold thioesterase	U

A0A5C5NGK2	Transcription termination/antitermination protein NusA	1.8	A0A5C5NDB4	ATP-dependent protease	U
A0A5C5NPA5	D-alanine--D-alanine ligase	1.8	A0A5C5NZ30	Protein-export chaperone SecB	U
A0A5C5N9H3	Elongation factor Tu	1.8	A0A5C5NGU5	(2Fe-2S)-binding protein	U
A0A5C5NS52	2-isopropylmalate synthase	1.7	A0A5C5P285	Response regulator	U
A0A5C5NKQ1	Molybdenum cofactor biosynthesis protein B	1.7	A0A5C5NFX3	Ribosome assembly RNA-binding protein YhbY	U
A0A5C5NPZ0	Extracellular solute-binding protein	1.7	A0A5C5NX13	DUF1508 domain-containing protein	U

A0A5C5NRQ9	FKBP-type peptidyl-prolyl cis-trans isomerase	1.7	A0A5C5NQC9	Hydroxyacylglutathione hydrolase	U
A0A5C5NT19	1.4-alpha-glucan branching protein GlgB	1.7	A0A5C5NVI2	HAD-IA family hydrolase	U
A0A5C5NV21	Pyrimidine/purine nucleoside phosphorylase	1.7	A0A5C5NFK4	DUF4347 domain-containing protein	U
A0A5C5NV30	3-oxoadipate enol-lactonase	1.7	A0A5C5NCI1	Acyl-CoA thioesterase II	U
A0A5C5NS02	YebC/PmpR family DNA-binding transcriptional regulator	1.6	A0A5C5N907	DUF411 domain-containing protein	U
A0A5C5N989	Preprotein translocase subunit SecY	1.6	A0A5C5NJR6	Chemotaxis protein CheA	U

A0A5C5NFF6	ABC transporter substrate-binding protein	1.6	A0A5C5NPX0	RNA polymerase factor sigma-54	U
A0A5C5P1E3	DNA-directed RNA polymerase subunit omega	1.6	A0A5C5NDI9	HlyC/CorC family transporter	U
A0A5C5NU34	Histidine--tRNA ligase	1.6	A0A5C5NQW3	Sugar kinase	U
A0A5C5NP41	Succinate dehydrogenase. cytochrome b556 subunit	1.6	A0A5C5NTK9	Agmatinase	U
A0A5C5NJ29	LOG family protein	1.6	A0A5C5NH77	Curli production assembly protein CsgG	U
A0A5C5N9T9	50S ribosomal protein L16	1.6	A0A5C5NVL1	ATPase	U

A0A5C5NDA4	50S ribosomal protein L17	1.5	A0A5C5NXK0	Acetylornithine deacetylase	U
A0A5C5NRG4	Quinolinate synthase NadA	1.5	A0A5C5NZC6	Acetyl-CoA C-acetyltransferase	U
A0A5C5NQM6	SDR family oxidoreductase	1.5	A0A5C5NZN4	23S rRNA (Adenine(2030)-N(6))-methyltransferase RlmJ	U
A0A5C5NVG0	Molybdopterin-synthase adenylyltransferase MoeB	1.5	A0A5C5NXV5	2-methylaconitate cis-trans isomerase PrpF	U
A0A5C5NWB6	Type I methionyl aminopeptidase	1.5	A0A5C5NLH7	FimV family protein	U

A0A5C5NYN0	Acetyl-CoA carboxylase biotin carboxylase subunit	1.5	A0A5C5NTJ1	Sorbosone dehydrogenase family protein	U
A0A5C5NPR2	Cell division protein FtsZ	1.5	A0A5C5N934	Cysteine hydrolase	U
A0A5C5NQR7	Nitrite/sulfite reductase	1.5	A0A5C5N819	Gamma-glutamyltransferase	U
A0A5C5N9C8	30S ribosomal protein S11	1.5	A0A5C5NSV1	TonB-dependent hemoglobin/transferrin/lactoferrin family receptor	U
A0A5C5NWE8	Valine--tRNA ligase	1.5	A0A5C5NJ90	CbbQ/NirQ/NorQ/GpvN family protein	U
A0A5C5NN86	Type III PLP-dependent enzyme	1.5	A0A5C5NAP9	Acyl-CoA dehydrogenase	U

A0A5C5NLW1	Enoyl-CoA hydratase	1.5	A0A5C5NRV3	LPS export ABC transporter ATP-binding protein	U
A0A5C5NW36	Uncharacterized protein	1.5	A0A5C5NU60	GAF domain-containing protein	U
A0A5C5N986	Outer membrane protein OmpW	1.5	A0A5C5NTV8	Kinase/pyrophosphorylase	U
A0A5C5NDD3	30S ribosomal protein S7	1.5	A0A5C5N9P7	XRE family transcriptional regulator	U
A0A5C5NK53	Molecular chaperone DnaJ	1.4	A0A5C5NDW9	Outer membrane lipoprotein chaperone LolA	U

A0A5C5NB91	Membrane protein insertase YidC	1.4	A0A5C5NL33	NADPH-dependent 2.4-dienoyl-CoA reductase	U
A0A5C5NT26	ADP-forming succinate--CoA ligase subunit beta	1.4	A0A5C5NR65	C-type cytochrome	U
A0A5C5NRY6	Bifunctional glutamate N-acetyltransferase/amino-acid acetyltransferase ArgJ	1.4	A0A5C5NTQ5	Uncharacterized protein	U
A0A5C5NVJ8	OmpA family protein	1.4	A0A5C5NYS5	Mechanosensitive channel MscK	U
A0A5C5NWZ0	Branched-chain amino acid ABC transporter substrate-binding protein	1.4	A0A5C5NQP7	3D-(3.5/4)-trihydroxycyclohexane-	U

				1.2-dione acylhydrolase (Decyclizing)	
A0A5C5NTX3	Chaperonin GroEL	1.4	A0A5C5NP14	C-type cytochrome biogenesis protein CcmI	U
A0A5C5NNY4	HTH-type transcriptional regulator CysB	1.4	A0A5C5NJX3	Creatininase family protein	U
A0A5C5NG01	Polynucleotide adenylyltransferase PcnB	1.4	A0A5C5P0K4	Glucans biosynthesis glucosyltransferase MdoH	U
A0A5C5NDD1	Acyl-CoA dehydrogenase	1.4	A0A5C5NDR1	Ethanolamine ammonia-lyase subunit EutC	U
A0A5C5P422	Dihydrolipoyllysine-residue acetyltransferase	1.4	A0A5C5NFG5	3-deoxy-manno-octulosonate cytidylyltransferase	U

A0A5C5N9R9	CoA transferase subunit A	1.3	A0A5C5NRP3	EAL domain-containing protein	U
A0A5C5NVW3	YbaB/EbfC family nucleoid-associated protein	1.3	A0A5C5NR73	DNA topoisomerase IB	U
A0A5C5NTU5	Threonylcarbamoyl-AMP synthase	1.3	A0A5C5NK14	PAS domain S-box protein	U
A0A5C5NXQ3	NADP-dependent oxidoreductase	1.3	A0A5C5NSD5	Methyl-accepting chemotaxis protein	U
A0A5C5NZG5	Peptide chain release factor 2	1.3	A0A5C5N8X5	DUF2934 domain-containing protein	U
A0A5C5NG00	Acetolactate synthase small subunit	1.3	A0A5C5NQN5	DUF3203 family protein	U

A0A5C5NC72	DUF1993 domain-containing protein	1.3	A0A5C5NP15	Uncharacterized protein	U
A0A5C5NYV6	RNA polymerase sigma factor RpoE	1.3	A0A5C5NP49	DUF883 domain-containing protein	U
A0A5C5NCV9	F0F1 ATP synthase subunit beta	1.3	A0A5C5N9D9	30S ribosomal protein S14	U
A0A5C5N9D2	30S ribosomal protein S19	1.3	A0A5C5NUB9	DUF2789 domain-containing protein	U
A0A5C5NZL9	YceI family protein	1.3	A0A5C5NBS7	Uncharacterized protein	U
A0A5C5NS58	23S rRNA (Adenine(2503)-C(2))-methyltransferase RlmN	1.3	A0A5C5NKM8	Uncharacterized protein	U

A0A5C5NDP1	Pyrroloquinoline-quinone synthase PqqC	1.3	A0A5C5NXZ2	Uncharacterized protein	U
A0A5C5NWQ9	Phosphate ABC transporter ATP-binding protein	1.3	A0A5C5N9F8	50S ribosomal protein L30	U
A0A5C5NKZ6	Trigger factor	1.3	A0A5C5NRJ4	Peptidase M4	U
A0A5C5NHN5	YciK family oxidoreductase	1.3	A0A5C5N839	Vanillate O-demethylase oxidoreductase VanB	U
A0A5C5NC34	Cytosine deaminase	1.3	A0A5C5NXC5	DUF1090 domain-containing protein	U
			A0A5C5N7Z9	RcnB family protein	U

		A0A5C5NZL8	Zinc-binding alcohol dehydrogenase family protein	U
		A0A5C5NMZ4	Stringent starvation protein A	U
		A0A5C5NHG8	Preprotein translocase subunit SecG	U
		A0A5C5NX28	Phosphate ABC transporter permease PstA	U
		A0A5C5NXF8	Penicillin-binding protein activator LpoB	U
		A0A5C5NTC5	Response regulator	U

		A0A5C5NVV3	Uncharacterized protein	U
		A0A5C5NLV2	Carbon storage regulator CsrA	U
		A0A5C5NTX1	Protein TolR	U
		A0A5C5NH80	Uncharacterized protein	U
		A0A5C5N951	Endonuclease/exonuclease/phosphatase family protein	U
		A0A5C5NFD2	Arc family DNA-binding protein	U
		A0A5C5NUJ3	Glutathione peroxidase	U

		A0A5C5ND27	GNAT family N-acetyltransferase	U
		A0A5C5NGV8	Large-conductance mechanosensitive channel protein MscL	U
		A0A5C5N8C5	Uncharacterized protein	U
		A0A5C5NWW8	AdeC/AdeK/OprM family multidrug efflux complex outer membrane factor	U
		A0A5C5NW10	DUF3509 domain-containing protein	U
		A0A5C5NUD0	RNA polymerase sigma factor FliA	U

		A0A5C5NDQ5	DNA-3-methyladenine glycosylase	U
		A0A5C5NZP5	Phosphate starvation-inducible protein PsiF	U
		A0A5C5NTB9	50S ribosomal protein L21	U
		A0A5C5NWX9	DUF3617 domain-containing protein	U
		A0A5C5NDF4	Neutral zinc metallopeptidase	U
		A0A5C5N800	Uncharacterized protein	U
		A0A5C5NVC5	Cell division protein ZipA	U

		A0A5C5NC51	DUF480 domain-containing protein	U
		A0A5C5NGU6	ATP-dependent RNA helicase RhlB	U
		A0A5C5N7K8	Ribonucleotide-diphosphate reductase subunit beta (Fragment)	U
		A0A5C5NR80	Arsenate reductase ArsC	U
		A0A5C5NZJ2	GNAT family N-acetyltransferase	U
		A0A5C5NCZ0	DNA repair protein RadA	U

		A0A5C5NPU3	Cell division protein ZapE	U
		A0A5C5NV97	Pyridoxamine 5'-phosphate oxidase	U
		A0A5C5NNQ2	BON domain-containing protein	U
		A0A5C5ND60	PilZ domain-containing protein	U
		A0A5C5NGI9	Amino acid deaminase	U
		A0A5C5NAT5	Co2+/Mg2+ efflux protein ApaG	U
		A0A5C5NNP1	Integrase	U

		A0A5C5NKP3	Nitrate reductase subunit beta	U
		A0A5C5NQZ4	N-acetyltransferase	U
		A0A5C5NR39	UPF0149 family protein	U
		A0A5C5NK57	Uncharacterized protein	U
		A0A5C5NN58	Cytochrome bc complex cytochrome b subunit	U
		A0A5C5P198	RNA chaperone Hfq	U
		A0A5C5NDR5	Glucose 1-dehydrogenase	U
		A0A5C5NNS9	Response regulator	U

		A0A5C5NQN9	GAF domain-containing protein	U
		A0A5C5NWS3	Enoyl-CoA hydratase/isomerase family protein	U
		A0A5C5NTJ8	Mechanosensitive ion channel	U
		A0A5C5NFU5	Tetratricopeptide repeat protein	U
		A0A5C5NEQ5	Thiosulfate sulfurtransferase GlpE	U

		A0A5C5NST5	Fe-S biogenesis protein NfuA	U
		A0A5C5NZ17	ParA family protein	U
		A0A5C5NUT8	Recombination protein RecR	U
		A0A5C5NGL2	Excinuclease ABC subunit A	U
		A0A5C5NJ58	Uncharacterized protein	U
		A0A5C5N9M1	(2E.6E)-farnesyl diphosphate synthase	U
		A0A5C5P2C7	Acyl-CoA thioesterase	U

		A0A5C5NE53	tRNA (N6-isopentenyl adenosine(37)-C2)-methylthiotransferase MiaB	U
		A0A5C5NU51	Preprotein translocase subunit YajC	U
		A0A5C5N7L2	DUF86 domain-containing protein	U
		A0A5C5NRE7	Thiamine pyrophosphate-dependent dehydrogenase E1 component subunit alpha	U

		A0A5C5NQL6	MurR/RpiR family transcriptional regulator	U
		A0A5C5NVE3	ATP-dependent RNA helicase HrpA	U
		A0A5C5NVX7	Biliverdin-producing heme oxygenase	U
		A0A5C5NVP6	3-hydroxyacyl-CoA dehydrogenase	U
		A0A5C5NZ90	AMIN domain-containing protein	U
		A0A5C5NWM5	Accessory factor UbiK family protein	U

		A0A5C5NA03	Nuclear transport factor 2 family protein	U
		A0A5C5NW14	PadR family transcriptional regulator	U
		A0A5C5NAC8	Helix-turn-helix transcriptional regulator	U
		A0A5C5NFV7	Glutathione S-transferase family protein	U
		A0A5C5NNY0	ABC transporter ATP-binding protein/permease	U
		A0A5C5NVW6	Tripartite tricarboxylate transporter substrate binding protein	U

		A0A5C5NEE3	Protein-glutamate O-methyltransferase CheR	U
		A0A5C5P3G5	Serine acetyltransferase	U
		A0A5C5NMV1	Outer membrane lipid asymmetry maintenance protein MlaD	U
		A0A5C5NYP4	Serine/threonine-protein phosphatase	U
		A0A5C5NUC0	Lipid kinase YegS	U
		A0A5C5NSK5	M23 family metallopeptidase	U

		A0A5C5P1V9	DUF3298 domain-containing protein	U
		A0A5C5NRJ9	Methyl-accepting chemotaxis protein	U
		A0A5C5NZX1	Amino acid ABC transporter substrate-binding protein	U
		A0A5C5NDY0	Uroporphyrinogen-III C-methyltransferase	U
		A0A5C5NVF1	Methyl-accepting chemotaxis protein	U
		A0A5C5NEM2	Uncharacterized protein	U

		A0A5C5NFX5	50S ribosomal protein L32	U
		A0A5C5NBD6	Peptidylprolyl isomerase	U
		A0A5C5NUQ9	DUF2892 domain-containing protein	U
		A0A5C5NS94	PepSY domain-containing protein	U
		A0A5C5NSV8	Uncharacterized protein	U
		A0A5C5NQN8	SRPBCC family protein	U
		A0A5C5NUX6	2-methylcitrate dehydratase	U
		A0A5C5P178	Esterase	U

		A0A5C5NI36	Translation initiation factor IF-1	U
		A0A5C5NZD0	TIGR02647 family protein	U
		A0A5C5P116	3-oxoacyl-ACP reductase FabG	U
		A0A5C5NRX8	Glycine cleavage system protein GcvH	U
		A0A5C5NTB2	Uncharacterized protein	U
		A0A5C5NW75	OmpA family protein	U
		A0A5C5NNW4	Alpha/beta hydrolase	U

		A0A5C5NU35	Response regulator transcription factor	U
		A0A5C5NWF0	Uncharacterized protein	U
		A0A5C5NYV7	Twin-arginine translocase TatA/TatE family subunit	U
		A0A5C5P3P7	Rhodanese-like domain-containing protein	U
		A0A5C5NEN6	Flagellar biosynthesis anti-sigma factor FlgM	U
		A0A5C5NY79	ATP-dependent RNA helicase DbpA	U

		A0A5C5P0N7	ATP-dependent protease subunit HslV	U
		A0A5C5NTR6	HIT domain-containing protein	U
		A0A5C5NEU0	DUF805 domain-containing protein	U
		A0A5C5NXT4	Flagellar basal body-associated protein FliL	U
		A0A5C5NH43	YheV family putative metal-binding protein	U
		A0A5C5NDN7	Pyrroloquinoline quinone biosynthesis protein PqqE	U

		A0A5C5N8A8	CHAD domain-containing protein	U
		A0A5C5NV62	Ferredoxin family protein	U
		A0A5C5NXY5	16S rRNA (Uracil(1498)-N(3))-methyltransferase	U
		A0A5C5N7Z5	Uncharacterized protein	U
		A0A5C5NVY2	Methyl-accepting chemotaxis protein	U
		A0A5C5NXB3	Ribose-5-phosphate isomerase RpiA	U
		A0A5C5NK07	Bifunctional diguanylate cyclase/phosphodiesterase	U

		A0A5C5NXC6	DUF1456 family protein	U
		A0A5C5NWU4	Energy transducer TonB	U
		A0A5C5N7N6	Uncharacterized protein	U
		A0A5C5NUI9	Alkylphosphonate utilization protein	U
		A0A5C5NK45	Ribosome maturation factor RimP	U
		A0A5C5NS00	tRNA preQ1(34) S-adenosylmethionine ribosyltransferase-isomerase QueA	U

		A0A5C5NEM8	Carbon storage regulator CsrA	U
		A0A5C5P070	Adenosine deaminase	U
		A0A5C5NYY0	RidA family protein	U
		A0A5C5NS76	Arylformamidase	U
		A0A5C5NR89	DUF2025 family protein	U
		A0A5C5NM56	Uncharacterized protein	U
		A0A5C5NSB7	DUF1244 domain-containing protein	U
		A0A5C5P4H6	Type II 3-dehydroquinate dehydratase	U

		A0A5C5NS69	DUF1311 domain-containing protein	U
		A0A5C5NJ56	Cytochrome c	U
		A0A5C5NJD8	Uncharacterized protein	U
		A0A5C5NVC8	2-C-methyl-D-erythritol 2.4-cyclodiphosphate synthase	U
		A0A5C5NZR6	Transcriptional regulator BetI	U
		A0A5C5NCA5	Efflux RND transporter periplasmic adaptor subunit	U

		A0A5C5P2A3	Heavy-metal-associated domain-containing protein	U
		A0A5C5ND00	Transcription elongation factor GreAB	U
		A0A5C5NWV0	Ribonuclease PH	U
		A0A5C5NE41	Uncharacterized protein	U
		A0A5C5NX04	Beta-agarase	U
		A0A5C5NXS2	Hydrocarbon binding protein	U
		A0A5C5NVH3	Cold-shock protein	U
		A0A5C5NXP7	Uncharacterized protein	U

		A0A5C5NK46	DNA/RNA non-specific endonuclease	U
		A0A5C5NAL2	Uncharacterized protein	U
		A0A5C5NMV4	KpsF/GutQ family sugar-phosphate isomerase	U
		A0A5C5NPY4	Dephospho-CoA kinase	U
		A0A5C5NIK1	L-threonylcarbamoyladenylate synthase	U
		A0A5C5NZF9	Amino acid ABC transporter permease	U
		A0A5C5NT64	tRNA (Cytosine(32)/uridine(32)	U

			-2'-O)-methyltransferase TrmJ	
		A0A5C5NSX2	Carbon-nitrogen hydrolase family protein	U
		A0A5C5NTH4	Chromosome segregation protein SMC	U
		A0A5C5ND61	Exodeoxyribonuclease VII small subunit	U
		A0A5C5NPY3	Zinc ABC transporter substrate-binding protein	U
		A0A5C5NN30	LysR family transcriptional regulator	U

		A0A5C5NLV5	Uncharacterized protein	U
		A0A5C5NVY4	Guanine deaminase	U
		A0A5C5NJM5	GntR family transcriptional regulator	U
		A0A5C5NA39	Deferrochelatase/peroxidase EfeB	U
		A0A5C5N7Y5	Glutamine synthetase	U
		A0A5C5NRV6	Mannitol dehydrogenase family protein	U
		A0A5C5NS25	Glycosyltransferase family 1 protein	U

		A0A5C5NVI7	Flagellar motor switch protein FliN	U
		A0A5C5NQ56	Purine nucleoside permease	U
		A0A5C5NIJ4	Purine-binding chemotaxis protein CheW	U
		A0A5C5NWZ5	DUF2288 domain-containing protein	U
		A0A5C5NWC4	Flavodoxin	U
		A0A5C5NJP5	Beta-lactamase	U
		A0A5C5NSI3	AGE family epimerase/isomerase	U

		A0A5C5NUL8	Uncharacterized protein	U
		A0A5C5NQG1	Uncharacterized protein	U
		A0A5C5NU89	Carbohydrate porin	U
		A0A5C5NTQ4	Cytochrome-c oxidase. cbb3-type subunit I	U
		A0A5C5NXG4	Choline ABC transporter substrate-binding protein	U
		A0A5C5NRB7	Isochorismatase family protein	U
		A0A5C5NIE5	Chemotaxis protein	U
		A0A5C5NCX0	TIM barrel protein	U

		A0A5C5NIH1	DUF1285 domain-containing protein	U
		A0A5C5NX41	Acyl-CoA thioesterase	U
		A0A5C5NE85	Secretin	U
		A0A5C5NHB3	Outer membrane assembly lipoprotein YfiO	U
		A0A5C5NEN1	TetR/AcrR family transcriptional regulator	U
		A0A5C5NVR0	Ribonuclease D	U
		A0A5C5NLR4	50S ribosomal protein L20	U

		A0A5C5NGA2	Response regulator	U
		A0A5C5NPP5	ATP-binding cassette domain-containing protein	U
		A0A5C5NK31	Uncharacterized protein	U
		A0A5C5NQN4	GAF domain-containing protein	U
		A0A5C5P4A9	Nicotinate phosphoribosyltransferase	U
		A0A5C5NEN2	NAD(P)H:quinone oxidoreductase	U

		A0A5C5NE00	tRNA 2-thiouridine(34) synthase MnmA	U
		A0A5C5P065	Serine protein kinase RIO	U
		A0A5C5NZE3	Outer membrane protein assembly factor BamE	U
		A0A5C5NL56	Zinc ABC transporter substrate-binding protein	U
		A0A5C5NES0	DUF3530 family protein	U
		A0A5C5NMZ5	Ribosome-associated protein	U

		A0A5C5NB15	Transcription antitermination factor NusB	U
		A0A5C5NUI2	DUF3509 domain-containing protein	U
		A0A5C5NTM8	Transcription elongation factor GreB	U
		A0A5C5NWZ9	Transcriptional regulator HexR	U
		A0A5C5P3R5	Histidine utilization repressor	U
		A0A5C5NM37	Response regulator transcription factor	U

		A0A5C5NUC1	Cytochrome-c oxidase. cbb3-type subunit II	U

Accession: Uniprot database identification; FC: Fold change; U: Unique protein

Supplementary Table S2. Identified Metabolic pathways for strain *Pseudomonas* sp. C9.

Up regulated		Down regulated		IPR treatment*	
**M. Pathway****	**Nº**	**M. Pathway****	**Nº**	**M. Pathway****	**Nº**
Total	76	Total	32	Total	81
Biosynthesis of secondary metabolites	29	Biosynthesis of secondary metabolites	2	Biosynthesis of secondary metabolites	29
Ribosome	22	Biosynthesis of cofactors	2	Two-component system	21
Microbial metabolism in diverse environments	20	Glutathione metabolism	2	Microbial metabolism in diverse environments	20
Carbon metabolism	16	Bacterial secretion system	2	Biosynthesis of amino acids	13
Biosynthesis of amino acids	12	Butanoate metabolism	2	Biosynthesis of cofactors	11
Quorum sensing	6	Biosynthesis of nucleotide sugars	2	Carbon metabolism	9
Citrate cycle (TCA cycle)	6	Oxidative phosphorylation	2	Ribosome	8
Pyruvate metabolism	6	Two-component system	2	ABC transporters	8
Glycolysis / Gluconeogenesis	6	Biofilm formation	2	Quorum sensing	6
Purine metabolism	6	O-Antigen nucleotide sugar biosynthesis	1	Biofilm formation	6
ABC transporters	5	Nucleotide excision repair	1	Butanoate metabolism	6
2-Oxocarboxylic acid metabolism	5	Styrene degradation	1	Phenylalanine. tyrosine. and tryptophan biosynthesis	5
Oxidative phosphorylation	5	Biotin metabolism	1	Glyoxylate and dicarboxylate metabolism	5
Nucleotide metabolism	5	Fatty acid biosynthesis	1	Glycine. serine. and threonine metabolism	5
Butanoate metabolism	5	Microbial metabolism in diverse environments	1	Pyruvate metabolism	5
Bacterial secretion system	4	Quorum sensing	1	Pentose phosphate pathway	5
RNA degradation	4	Protein export	1	Flagellar assembly	4
Methane metabolism	4	Tyrosine metabolism	1	Bacterial chemotaxis	4
Pyrimidine metabolism	4	Fatty acid metabolism	1	Glutathione metabolism	4
Pentose phosphate pathway	4	Cyanoamino acid metabolism	1	Oxidative phosphorylation	4

Glyoxylate and dicarboxylate metabolism	4	Starch and sucrose metabolism	1	Fatty acid metabolism	4
Protein export	3	D-Amino acid metabolism	1	beta-Lactam resistance	4
Glycine. serine and threonine metabolism	3	Pentose phosphate pathway	1	Protein export	4
Starch and sucrose metabolism	3	Biosynthesis of various plant secondary metabolites	1	Bacterial secretion system	4
Propanoate metabolism	3	Phenylalanine metabolism	1	RNA degradation	4
Tryptophan metabolism	3	Pentose and glucuronate interconversions	1	Alanine. aspartate. and glutamate metabolism	3
Biosynthesis of cofactors	2	Degradation of flavonoids	1	Cysteine and methionine metabolism	3
Glutathione metabolism	2	Glyoxylate and dicarboxylate metabolism	1	Nitrogen metabolism	3
Lysine biosynthesis	2	Ascorbate and aldarate metabolism	1	Fatty acid degradation	3
Sulfur relay system	2	Nicotinate and nicotinamide metabolism	1	Nucleotide metabolism	3
One carbon pool by folate	2	Bacterial chemotaxis	1	Purine metabolism	3
C5-Branched dibasic acid	2	RNA degradation	1	Terpenoid backbone biosynthesis	3
Homologous recombination	2			Propanoate metabolism	3
RNA polymerase	2			Pyrimidine metabolism	3
Lipopolysaccharide biosynthesis	2			Cationic antimicrobial peptide (CAMP) resistance	3
Arginine and proline metabolism	2			Starch and sucrose metabolism	3
Sulfur metabolism	2			Benzoate degradation	2
Aminoacyl-tRNA biosynthesis	2			Valine. leucine. and isoleucine degradation	2
Alanine. aspartate. and glutamate metabolism	2			Sulfur metabolism	2
Valine. leucine. and isoleucine biosynthesis	2			Arginine biosynthesis	2
Peptidoglycan biosynthesis	2			Biosynthesis of nucleotide sugars	2
Fructose and mannose metabolism	2			Lysine degradation	2
Benzoate degradation	2			Ubiquinone and other terpenoid-quinone biosynthesis	2

Arginine biosynthesis	2
Valine. leucine. and isoleucine degradation	2
Lysine degradation	2
Folate biosynthesis	1
Lipoic acid metabolism	1
D-Amino acid metabolism	1
Nicotinate and nicotinamide metabolism	1
Histidine metabolism	1
DNA replication	1
Degradation of aromatic compounds	1
Biosynthesis of nucleotide sugars	1
Vancomycin resistance	1
Biofilm formation	1
Two-component system	1
Fatty acid metabolism	1
Efferocytosis	1
Chloroalkane and chloroalkene degradation	1
Pantothenate and CoA biosynthesis	1
Fatty acid biosynthesis	1
Phenylalanine. tyrosine. and tryptophan biosynthesis	1
Cyanoamino acid metabolism	1
Cysteine and methionine metabolism	1
Mismatch repair	1
Streptomycin biosynthesis	1
Galactose metabolism	1
Biosynthesis of nucleotide sugars	1

Porphyrin metabolism	2
Lipopolysaccharide biosynthesis	2
Nucleotide excision repair	2
Tryptophan metabolism	2
2-Oxocarboxylic acid metabolism	2
Mismatch repair	2
Base excision repair	2
Vitamin B6 metabolism	1
Fatty acid biosynthesis	1
Sulfur relay system	1
Exopolysaccharide biosynthesis	1
Non-homologous end-joining	1
Citrate cycle (TCA cycle)	1
beta-Alanine metabolism	1
Phenylalanine metabolism	1
Taurine and hypotaurine metabolism	1
One carbon pool by folate	1
Pantothenate and CoA biosynthesis	1
Homologous recombination	1
Selenocompound metabolism	1
Folate biosynthesis	1
Cyanoamino acid metabolism	1
Glycerophospholipid metabolism	1
Biosynthesis of unsaturated fatty acids	1
Biotin metabolism	1
Riboflavin metabolism	1

Amino sugar and nucleotide sugar metabolism	1		Nicotinate and nicotinamide metabolism	1
Phenylalanine metabolism	1		Lipoic acid metabolism	1
Fatty acid degradation	1		C5-Branched dibasic acid metabolism	1
Caprolactam degradation	1		DNA replication	1
Pinene. camphor and geraniol degradation	1		Porphyrin metabolism	1
Aminobenzoate degradation	1		Valine. leucine. and isoleucine biosynthesis	1
beta-Alanine metabolism	1		Carbapenem biosynthesis	1
			Arginine and proline metabolism	1
			Non-homologous end-joining	1
			Aminobenzoate degradation	1
			Styrene degradation	1
			Biosynthesis of various plant secondary metabolites	1

IPR Treatment*: Only expressed in IPR Treatment; M. Pathway**: Metabolic pathways by KEEG; N°: number of involved proteins.
